# Ramsay Hunt Syndrome Associated With Etanercept Treatment for Rheumatoid Arthritis: A Case Report and a Review of Literature

**DOI:** 10.7759/cureus.66531

**Published:** 2024-08-09

**Authors:** Sarah E Robinson, Rahul Varman

**Affiliations:** 1 Plastic and Reconstructive Surgery, Creighton University School of Medicine, Omaha, USA

**Keywords:** immunosuppression, new drug reaction, ramsay hunt syndrome (rhs), etanercept, rheumatoid arthritis

## Abstract

A 38-year-old woman with rheumatoid arthritis treated with etanercept presented with complaints of ear pain. Over four days, the pain progressed to a vesicular rash and then facial nerve paralysis. The patient was diagnosed with Ramsay Hunt syndrome (RHS), a reactivation of the varicella zoster virus that specifically affects the seventh cranial nerve (CN VII). Etanercept is an anti-tumor necrosis factor (anti-TNF) agent that has known immunosuppressive effects. RHS occurs more commonly in immunocompromised states, such as the one induced by etanercept. To the best of our knowledge, this is one of the first reported cases of RHS with etanercept treatment.

## Introduction

Etanercept is an anti-tumor necrosis factor (anti-TNF) agent that was among the first of its kind in the treatment of rheumatoid arthritis (RA). It is a recombinant fusion protein that targets TNF-α. It has been approved to treat RA since 1999 [1]. Etanercept carries a boxed warning as it places patients at a higher risk for developing infections that may lead to hospitalization or death [1]. Varicella zoster virus (VZV) is the virus responsible for causing the conditions varicella and herpes zoster. After initial infection, VZV remains in the dorsal root ganglion and can reactivate as herpes zoster which presents as a burning, vesicular rash along a dermatomal distribution [2]. Ramsay Hunt syndrome (RHS) is the result of an acute reactivation of VZV at the geniculate ganglion affecting the facial nerve (CN VII) and the vestibulocochlear nerve (CN VIII) [3]. The syndrome is characterized by herpes zoster oticus accompanied by facial nerve palsy, additional symptoms can include tinnitus and hearing loss [2]. The diagnosis of RHS is primarily clinical. Early identification is crucial, as antiviral therapy must be initiated within 72 hours of symptom onset to maximize recovery outcomes [4].

## Case presentation

A 38-year-old woman presented to her primary care physician with complaints of right ear pain and lymphadenopathy. The patient had been diagnosed with RA over six years ago and was being managed with weekly etanercept (50 mg/mL IM) and hydroxychloroquine (200 mg PO daily). Upon examination, a bulging, erythematous tympanic membrane was noted. The patient was diagnosed with otitis media and was prescribed amoxicillin 500 mg. At this time, the patient discontinued her etanercept.

**Figure 1:**
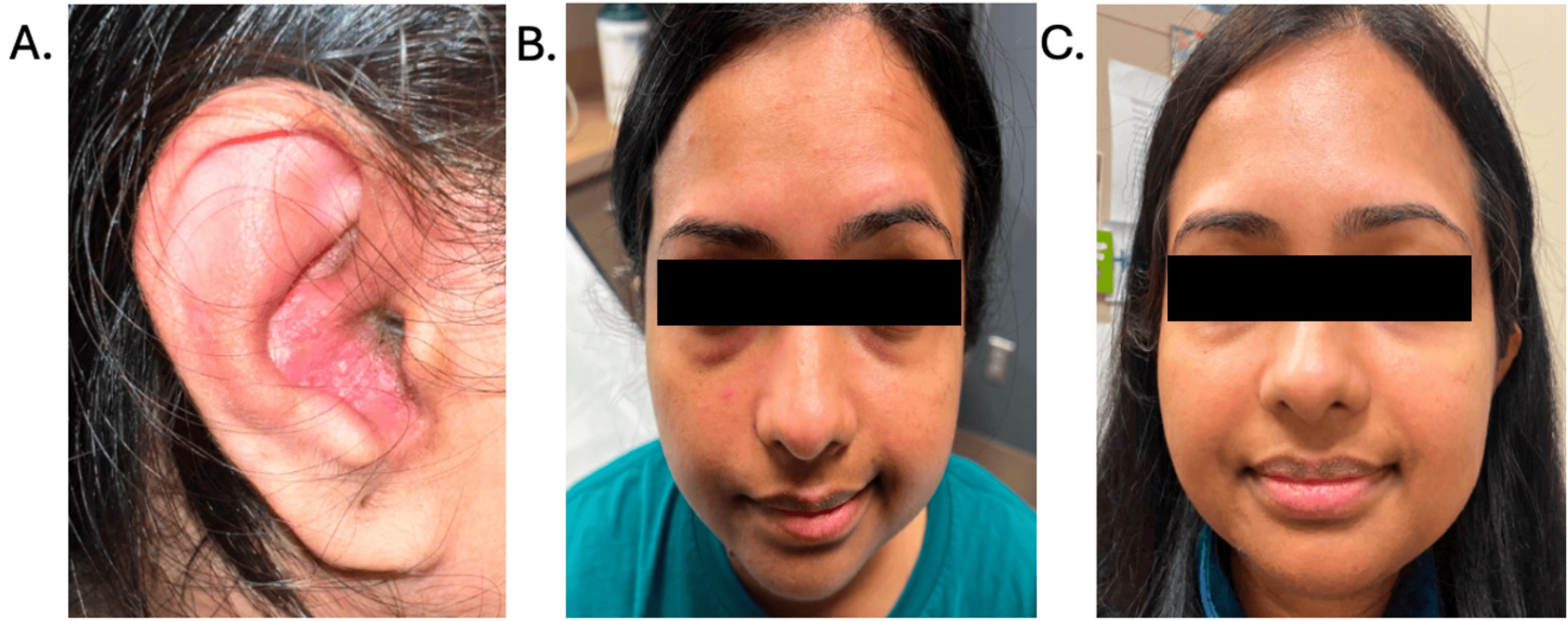
Patient Progression (A) Day 4: Ear redness, drainage, and auricular swelling are present. Some evidence of a vesicular rash is visible. (B) Photo taken in the emergency department (ED): Evaluation shows hypodynamic forehead and eyebrow movement, lagophthalmos, an effaced nasolabial fold, poor upper lip elevation, and a grossly asymmetric smile. (C) Approximately three months after the initial presentation: There is a restoration of gross facial symmetry, both during smiling and at rest (not pictured). The lagophthalmos has resolved.

Four days later, she returned with a new complaint of drainage from her right ear (Figure 1A). A physical exam showed swelling of the auditory canal and auricle with accompanied drainage and mastoid tenderness. The patient was prescribed ciprofloxacin 0.3%/dexamethasone 0.1% ear drops and was switched to a 10-day course of Augmentin.

Five days after her initial visit, she returned to the clinic with new onset right-side facial weakness. She was sent to the emergency department where they reported a vesicular rash of the right external ear and peripheral nerve palsy (Figure 1B). The patient was unable to raise her eyebrow, wrinkle her forehead, or raise her lip. At that time, she was empirically started on valacyclovir and prednisone for suspected RHS.

Otolaryngological examination the following day confirmed CN VII damage with flaccid paralysis of the right face including an effaced nasolabial fold and poor upper lip elevation with a smile. This further confirmed the RHS diagnosis. Lagophthalmos with poor protection of the cornea was noted on the exam and the patient was fitted with an eyelid weight. The patient was instructed to continue taking antivirals and prednisone and was referred to speech-language pathology for therapy.

Three months post RHS diagnosis, the patient has recovered bilateral facial symmetry at rest, with brow raise, and with eye closure and smile without synkinesis (Figure 1C).

## Discussion

RHS is the reactivation of VZV from the geniculate ganglion affecting CN VII. It is characterized by the combination of zoster oticus and peripheral facial nerve paralysis [3]. Diagnosis is generally made based on patient presentation, though titers for VZV load can also be drawn and assessed. Treatment for RHS involves administration of an antiviral, like acyclovir or valaciclovir, and oral corticosteroids [5]. Identification and treatment within the first 72 hours of symptom onset had a 75% complete recovery rate compared to only 30% complete recovery when treatment was started at or after day 7 [4]. These findings emphasize the importance of early intervention to optimize recovery outcomes and minimize long-term complications and deficits.

The development of biological drugs like etanercept has revolutionized the treatment of RA. Etanercept is a recombinant anti-TNF protein, and it has consistently shown a reduction in both symptoms and progression of RA [6]. Despite its clinical efficacy and overall safety, etanercept carries increased risks of infection related to its immunosuppressive function, including tuberculosis, *Staphylococcus aureus*, and VZV [7]. Previous research indicates there is an increased incidence of herpes zoster in patients receiving anti-TNF-α treatment for RA (P=0.01) [8]. However, there was no statistically significant increase observed in patients taking etanercept (P=0.14), specifically [9]. Cases have been reported of patients with concurrent RA and RHS while on infliximab and tocilizumab, TNF-α, and interleukin 6 (IL-6) inhibitory monoclonal antibodies, respectively [9,10]. A rare case of meningo-rhombencephalitis has been noted with etanercept [11]. RHS has also been observed in patients on biologics for other autoimmune conditions. These cases are summarized in Table 1.

**Table 1:** RHS Cases Associated With RA Biologic Treatment Modalities Summary of reports of RHS in patients being treated with biologic modalities that can be used to treat RA and other autoimmune conditions. RHS: Ramsay Hunt syndrome; RA: rheumatoid arthritis; TNF-α: tumor necrosis factor-alpha

Drug	Mechanism of Action	Patient’s Indication for Biologic	Reference
Adalimumab	TNF-α inhibitor	Crohn disease	[12]
Etanercept	TNF-α inhibitor	Rheumatoid arthritis	[11]
Infliximab	TNF-α inhibitor	Ulcerative colitis	[13]
Tocilizumab	Interleukin-6 inhibitor	Rheumatoid arthritis	[9]
Tofacitinib	Janus kinase inhibitor	Rheumatoid arthritis	[10]

While research indicates etanercept has decreased risk relative to monoclonal antibody TNF inhibitors, it is still important to be aware that RHS is possible in patients taking etanercept. Treating RHS within the first 72 hours of onset is critical for improving long-term outcomes and shortening the duration of symptoms [4]. As such, it is important to keep RHS in mind when examining a patient on TNF-α inhibitors like etanercept.

## Conclusions

RHS poses a significant risk to patients undergoing treatment with biologic drugs like etanercept for RA. While etanercept carries a decreased risk relative to other similar TNF inhibitors, clinicians must remain vigilant in recognizing and promptly treating RHS to optimize outcomes and mitigate long-term complications.
